# Highly lethal genotype I and II recombinant African swine fever viruses detected in pigs

**DOI:** 10.1038/s41467-023-38868-w

**Published:** 2023-05-29

**Authors:** Dongming Zhao, Encheng Sun, Lianyu Huang, Leilei Ding, Yuanmao Zhu, Jiwen Zhang, Dongdong Shen, Xianfeng Zhang, Zhenjiang Zhang, Tao Ren, Wan Wang, Fang Li, Xijun He, Zhigao Bu

**Affiliations:** 1 State Key Laboratory for Animal Disease Control and Prevention, National High Containment Facilities for Animal Diseases Control and Prevention, Harbin Veterinary Research Institute, Chinese Academy of Agricultural Sciences, Harbin, People’s Republic of China; 2 Jiangsu Co-innovation Center for Prevention and Control of Important Animal Infectious Diseases and Zoonoses, Yangzhou University, Yangzhou, People’s Republic of China

**Keywords:** Viral epidemiology, Viral pathogenesis

## Abstract

African swine fever virus (ASFV) poses a great threat to the global pig industry and food security. Currently, 24 ASFV genotypes have been reported but it is unclear whether recombination of different genotype viruses occurs in nature. In this study, we detect three recombinants of genotype I and II ASFVs in pigs in China. These recombinants are genetically similar and classified as genotype I according to their *B646L* gene, yet 10 discrete fragments accounting for over 56% of their genomes are derived from genotype II virus. Animal studies with one of the recombinant viruses indicate high lethality and transmissibility in pigs, and deletion of the virulence-related genes MGF_505/360 and EP402R derived from virulent genotype II virus highly attenuates its virulence. The live attenuated vaccine derived from genotype II ASFV is not protective against challenge of the recombinant virus. These naturally occurring recombinants of genotype I and II ASFVs have the potential to pose a challenge to the global pig industry.

## Abstract (web summary)

Potential recombination of African swine fever virus genotypes is not well understood. Here, Zhao et al. report naturally occurring ASFVs carrying mosaic genomes of genotype I and II and show that the recombinant virus is lethal in pigs and evades the immunity induced by genotype II live vaccine.

## Introduction

African swine fever (ASF) is a devastating infectious disease in swine that is a severe threat to the global pig industry (www.woah.org). ASF viruses (ASFVs) belong to the genus Asfivirus and Asfarviridae family^1,2^. The ASFV *B646L* gene encodes the viral capsid protein P72 that plays an essential role in virion assembly and virus attachment to the host cell^1,3^. ASFVs are divided into 24 genotypes based on the C-terminal sequence of their *B646L* gene with 86.2–99.5% nucleotide identity^4^. All 24 genotypes of ASFVs have been detected in sub-Saharan Africa, and only two of these genotypes have spread outside of Africa^2,5^. In 1957, genotype I ASFV was introduced into Europe and caused ASF outbreaks in many European countries^6^. In 2007, a highly lethal genotype II ASFV (Georgia07) appeared in Georgia, and quickly spread to neighboring countries (www.woah.org). In 2018, Georgia07-like ASFV spread to China and other Asian countries^7^, and has since been responsible for the loss of over seven million swine in Eurasian countries (www.woah.org).

Pigs are not vaccinated against ASF; therefore, due to the lack of an effective control strategy, ASFV is widespread and continues to evolve in many countries^8,9^. Since 2018, we have been actively monitoring the genetic and biological changes of the virus in China. In 2020, several variants of genotype II viruses with reduced pathogenicity in pigs were detected in China, and this reduced pathogenicity was attributed to mutations and deletions in their genomes that resulted in disruption of the expression of the CD2v protein, an important virulence factor of the genotype II ASFV^10,11^. We also found that low-virulent genotype I ASFVs emerged in pigs in China in 2021^12^, and that the strains had no hemadsorption (HAD-negative) and were genetically similar to the NH/P68 strain reported in Portugal in the 1960s^13^. Both genotype I and genotype II ASFVs have been found in pigs in China, so we asked: is it possible that these viruses recombine in nature? And, if so, what challenges will be posed by the recombinant strains?

Here, we isolated three recombinants of genotype I and II ASFVs in fields in China. The recombinants were fully characterized for their genomes, and their virulence and immune escape capability were tested in pigs. The results revealed the fast evolution of ASFVs through genomic recombination between different genotypes, which poses a new challenge to disease control efforts and vaccine development.

## Results

### Recombinant ASFVs with mosaic genomes of genotype I and II viruses detected from field samples in China

During our surveillance in China, we isolated three ASFVs from pig samples collected in Jiangsu province, Henan province, and Inner Mongolia Autonomous Region, respectively, and designated them Pig/Jiangsu/LG/2021 (JS/LG/21), Pig/Henan/123014/2022 (HeN/123014/22), and Pig/Inner Mongolia/DQDM/2022 (IM/DQDM/22), respectively. We identified these strains as genotype I according to their *B646L* gene sequence (Supplementary Fig. 1). However, we were surprised to find that these viruses are all HAD-positive (Supplementary Fig. 2), since the previously detected genotype I viruses in China have all been HAD-negative^12^. The HAD-positive phenotype of ASFV is mainly determined by the integrity of the CD2v protein, which is encoded by the *EP402R* gene^14^. We, therefore, sequenced the *EP402R* gene of these three viruses, and found that their *EP402R* genes share 100% identity with that of the HLJ/18 virus, the first genotype II isolate in China^15,16^, but only 81% identity with that of the genotype I virus SD/DY-I/21 detected in China^12^.

We then performed next-generation sequencing for the *B646L* and *EP402R* genes from the field samples as well as purified virus stocks. The PCR amplification for the *B646L* and *EP402R* genes were cloned into T-Vector pMD19, and 20 positive clones for each reaction were randomly picked for sequencing by using the Sanger sequencing method. The results indicate that the *B646L* gene and *EP402R* gene of these newly emerging viruses are from genotype I virus and genotype II virus, respectively.

**Fig. 1:**
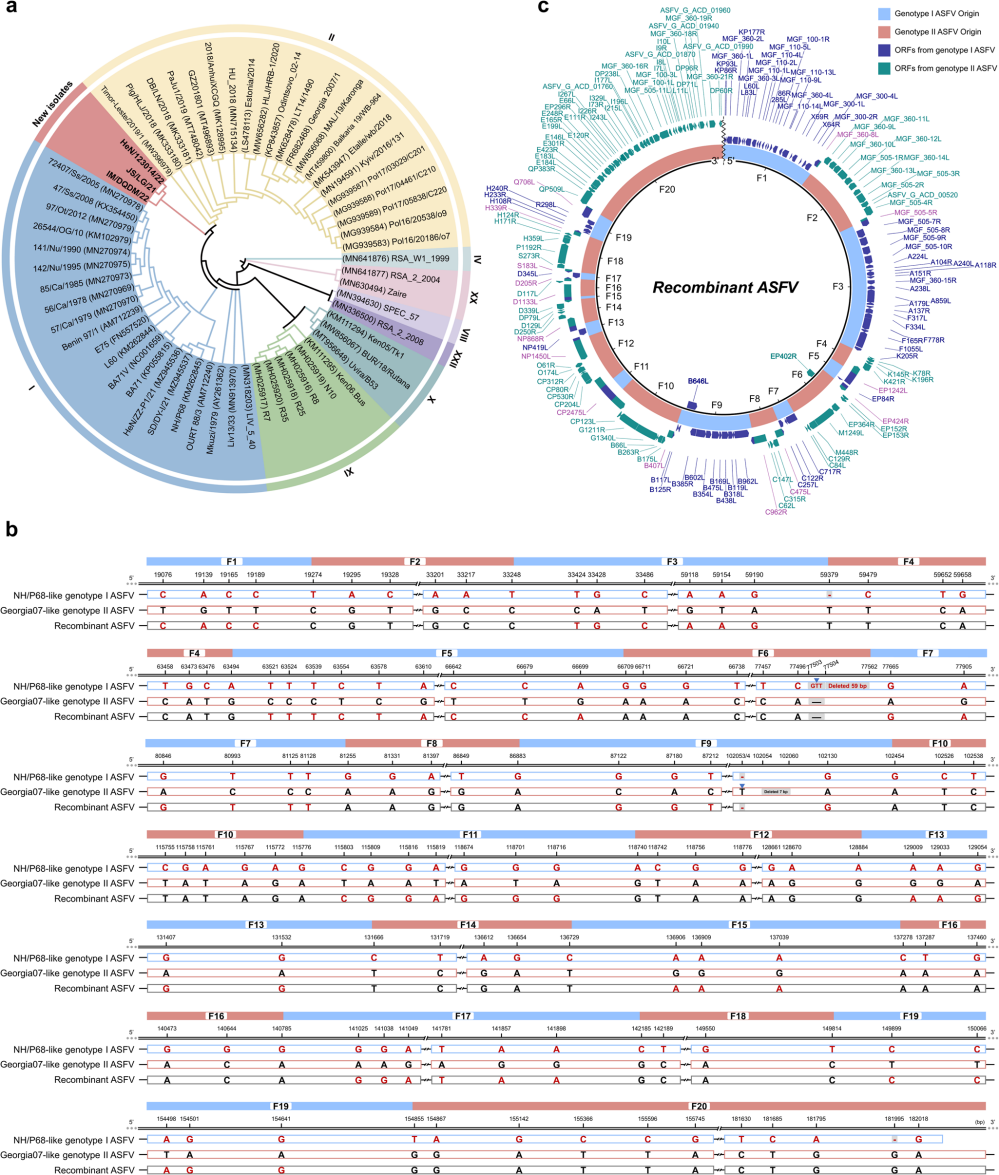
Genomic analysis of newly emerging recombinant African swine fever viruses. **a** The phylogenetic tree was built using the maximum likelihood (ML) and IQ-Tree based on the full genome sequences of the three recombinant ASFVs and 56 reference ASFVs of eight different genotypes from the GenBank database. **b** Specific single nucleotide polymorphisms (SNPs) at the junction regions of two recombinant fragments. The whole genomes of three recombinant ASFVs were compared with those of the representative NH/P68-like genotype I ASFV SD/DY-I/21 and Georgia07-like genotype II ASFV HLJ/18 by using SnapGene software. The recombinant fragments derived from genotype I ASFV are labeled in blue, and the fragments derived from genotype II ASFV in pink. **c** The diagram of the recombinant ASFV was created using BLAST Ring Image Generator with the whole genome sequence of JS/LG/21.

To further investigate the genomic composition of these viruses, we amplified and sequenced their whole genomes (GenBank accession numbers: OQ504954 for HeN/123014/22, OQ504955 for IM/DQDM/22, and OQ504956 for JS/LG/21) by using primers as described previously in ref. ^16^, and adjacent PCR amplification segments share an overlap of 100–200 base pairs (bps) to cover the sequences generated by the primers. We then performed a phylogenic analysis of the three viruses and 56 reference viruses of the eight different genotypes that are available in GenBank. The lengths of the genomes of JS/LG/21, HeN/123014/22, and IM/DQDM/22 are 185,431, 185,395, and 185,342 bps, respectively, and each of them has 172 open reading frames (ORFs). The three emerging viruses share 99.97–99.99% homology with each other at the nucleotide level, and form a unique branch in the phylogenic tree, which clusters between the genotype I and II viruses (Fig. 1a).

Georgia07-like genotype II and NH/P68-like genotype I ASFVs have been detected in fields in China^12,15–17^. Their respective representatives are genotype II virus HLJ/18 (GenBank: MK333180) and genotype I virus SD/DY-I/21 (GenBank: MZ945537), which share 97.38% nucleotide identity^12,16^. Compared with that of SD/DY-I/21, the genome of HLJ/18 has 21,062 bp insertions, 4237 bp deletions, and 3840 bp mutations. Both viruses have genotype-specific single nucleotide polymorphisms (SNPs) in the coding and noncoding regions of their genomes (Fig. 1b). To characterize the genomes of the three recombinant ASFVs, we performed a recombination analysis to determine the likely parents of the different fragments of the recombinants based on the specific SNPs of the genotype I and II ASFVs. A detailed comparison revealed that ten large discrete genome fragments of the new viruses are genotype I virus origin (Fig. 1c), as evidenced by the fact that these fragments have genotype I-specific SNPs (Fig. 1b), 99.36–100% identity with the homologous genome fragments of SD/DY-I/21, but 71.28–99.15% identity with those of HLJ/18 (Table 1 and Supplementary Table 1). The other ten discrete genome fragments of the new viruses are genotype II virus origin (Fig. 1c), as they have genotype II-specific SNPs (Fig. 1b), 99.95–100% identity with the homologous genome fragments of HLJ/18, but 27.50–98.32% identity with those of the SD/DY-I/21 (Table 1 and Supplementary Table 1).The total lengths of the ten fragments in the three emerging viruses that are derived from genotype I virus range from 80,648 to 80,737 bps, accounting for 43.51–43.54% of their genomes, whereas the total lengths of the ten fragments in the three emerging viruses that are derived from genotype II virus are 104,694 bps, accounting for 56.46–56.49% of their genomes (Table 1 and Supplementary Table 1). Of note, the genotype-determining gene *B646L* of all three emerging viruses is in fragment 9 (F9), which is from genotype I virus, whereas the *EP402R* gene encoding CD2v is in fragment 6 (F6), which is from genotype II virus (Fig. 1c); this explains why the novel genotype I viruses have the HAD-positive phenotype. Information about the recombinant fragments and all of the ORFs of the three recombinants are shown in Supplementary Dataset 1. These results thus demonstrate that the three emerging viruses are complicated recombinants with mosaic genomes of genotype I and genotype II ASFVs.

**Table 1:** Homology of each fragment of recombinant JS/LG/21 with the corresponding fragment of genotype I and genotype II African swine fever viruses

Fragment				Identity (%) with that of		Likely parent
ID	Position		Length (bp)	SD/DY-I/21 (Genotype I)	HLJ/18 (Genotype II)	
	Start	End				
F1	1	19,273	19,273	99.93	71.29	Genotype I
F2	19,274	33,248	13,975	27.50	99.99	Genotype II
F3	33,249	59,378	26,130	99.99	93.71	Genotype I
F4	59,379	63,494	4116	96.55	100.00	Genotype II
F5	63,495	66,708	3214	100.00	95.41	Genotype I
F6	66,709	77,562	10,854	89.57	100.00	Genotype II
F7	77,563	81,254	3692	99.89	97.49	Genotype I
F8	81,255	86,883	5629	97.00	100.00	Genotype II
F9	86,884	102,453	15,570	99.36	95.43	Genotype I
F10	102,454	115,776	13,323	93.73	100.00	Genotype II
F11	115,777	118,739	2963	100.00	90.15	Genotype I
F12	118,740	128,884	10,145	96.03	99.98	Genotype II
F13	128,885	131,665	2781	100.00	98.01	Genotype I
F14	131,666	136,729	5064	98.32	100.00	Genotype II
F15	136,730	137,277	548	100.00	98.91	Genotype I
F16	137,278	140,785	3508	97.97	100.00	Genotype II
F17	140,786	142,184	1399	99.71	99.15	Genotype I
F18	142,185	149,814	7630	97.03	100.00	Genotype II
F19	149,815	154,854	5040	100.00	98.21	Genotype I
F20	154,855	185,431	30,577	85.60	99.98	Genotype II

### The recombinants carry nucleotide mutations, insertions, and deletions in their genomes compared to their parent-like viruses

**Fig. 2:**
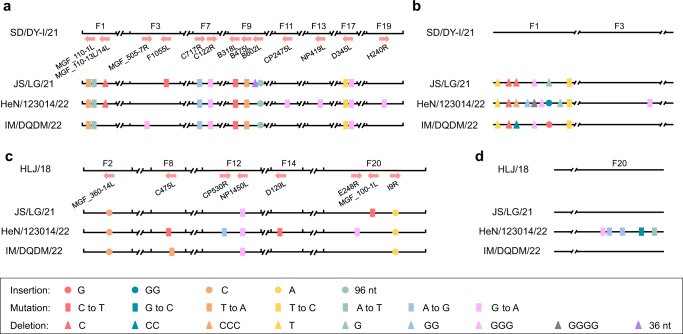
Genetic changes in the three recombinant ASFVs compared with genotype I and II ASFVs. Nucleotide mutations, deletions, and insertions in the three recombinant ASFVs compared with the corresponding regions of genotype I virus SD/DY-I/21 (**a**, **b**) and genotype II virus HLJ/18 (**c**, **d**) in the OFRs (**a**, **c**) and the noncoding regions (**b**, **d**).

Our previous study found that nucleotide mutations, insertions, and deletions have frequently occurred in ASFVs and some changes have resulted in alteration of the ORFs^11,12,16^. We, therefore, compared the genomes of the three emerging viruses with that of SD/DY-I/21 and HLJ/18^12,15,16^. Compared with homologous fragments of SD/DY-I/21, the three recombinants all have eight single nucleotide mutations in six ORFs in four different fragments, a 96-nucleotide fragment insertion in the ORF of *B602L*, and a single nucleotide mutation, two single nucleotide deletions, and a 3-nucleotide deletion in the noncoding region in F1 (Fig. 2a, b). JS/LG/21 and HeN/123014/22 both have a C deletion in *MGF_110-13/14L*. In addition, JS/LG/21 has a unique single nucleotide mutation and a 36-nucleotide deletion in the coding regions. HeN/123014/22 has three unique single nucleotide mutations in three ORFs, and IM/DQDM/22 has a single nucleotide mutation in the ORF of *MGF_505-7R* (Fig. 2a, b).

Compared with the homologous genomic sequence of HLJ/18, the three recombinants have the same single nucleotide mutation and two single nucleotide insertions in three ORFs (Fig. 2c). JS/LG/21 has a unique single nucleotide mutation in the ORF of *MGF_100-1L*. HeN/123014/22 has four unique single nucleotide mutations in four ORFs and five unique single nucleotide mutations in the noncoding regions in F20 (Fig. 2c, d). IM/DQDM/22 has a unique single nucleotide mutation in the ORF of *C475L* (Fig. 2c).

The nucleotide insertions and deletions in the genomic fragments derived from genotype I virus result in the alteration of one ORF of the recombinant IM/DQDM/22 virus and two ORFs of the recombinants JS/LG/21 and HeN/123014/22, compared with the SD/DY-I/2 virus (Supplementary Table 2), and the nucleotide insertions in the genomic fragments derived from genotype II virus result in the alteration of two ORFs of the three recombinant viruses, compared with the HLJ/18 virus (Supplementary Table 3).

### The recombinant virus JS/LG/21 is highly lethal and transmissible in pigs

The ten discrete fragments from highly virulent genotype II virus in the novel recombinant viruses bear 106 ORFs (Fig. 1 and Supplementary Dataset 1). Of note, some of these genes are virulence determinants, such as the *EP402R* gene and the six genes in the *MGF_505/360* region (*MGF_505-1R*, *MGF_505-2R*, *MGF_505-3R*, *MGF_360-12L*, *MGF_360-13L*, and *MGF_360-14L*) (we refer to these six genes as the “*MGF_505/360* genes” in this report)^11,18,19^. To investigate whether the genes from the lethal genotype II virus contribute to the pathogenicity and transmissibility of the recombinant ASFV, groups of six 7-week-old specific-pathogen-free (SPF) pigs were inoculated with 10^3^ HAD_50_ or 10^6^ HAD_50_ of JS/LG/21. Two uninoculated pigs were cohoused with each group of inoculated pigs to evaluate virus transmissibility. Pigs were monitored daily for clinical signs. Oral and rectal swabs and blood were collected from all of the pigs every other day starting on day 3 post-inoculation (p.i.) to detect viral shedding and viremia by qPCR.

**Fig. 3:**
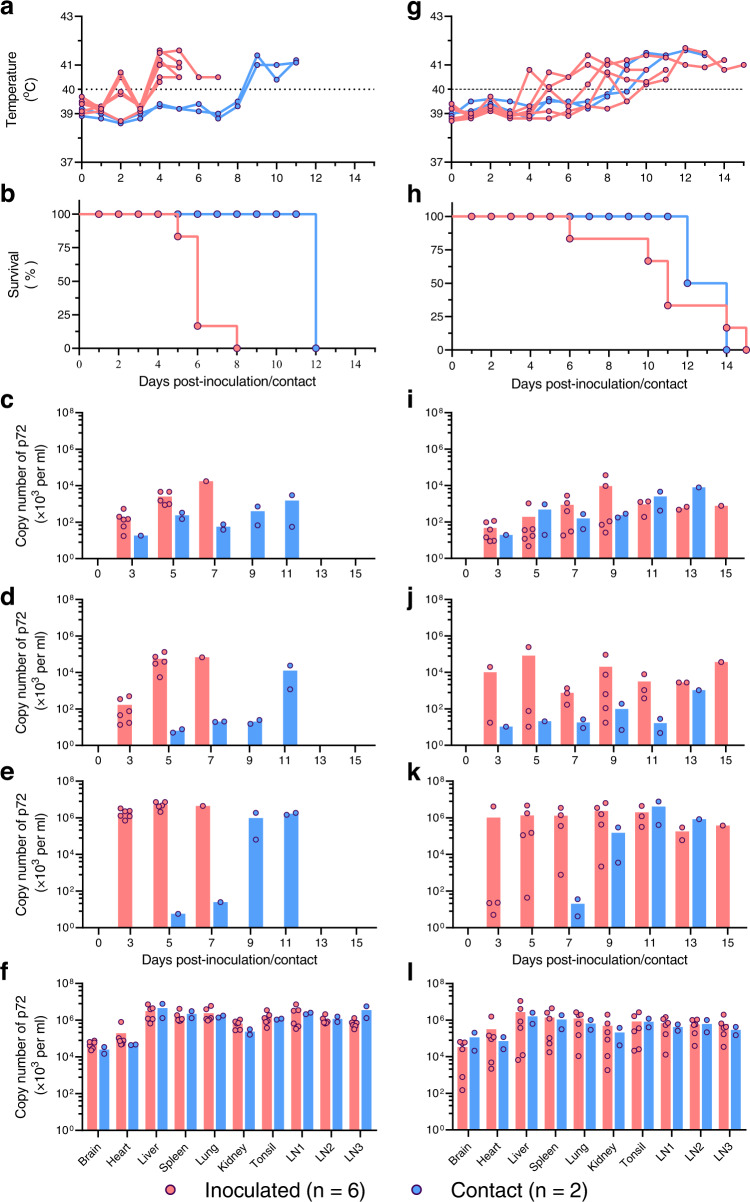
Pathogenicity and transmissibility of the recombinant African swine fever virus JS/LG/21 in pigs. Groups of six SPF pigs were inoculated with 10^6^ HAD_50_ (**a**–**f**) or 10^3^ HAD_50_ (**g**–**l**) of JS/LG/21, and two naive SPF pigs were cohoused with each group from the first day of inoculation. The rectal temperature (**a**, **g**) and survival of the pigs (**b**, **h**) were monitored daily. Oral swabs (**c**, **i**), rectal swabs (**d**, **j**), and blood (**e**, **k**) were collected at the indicated timepoints, and tissue samples (**f**, **l**) were collected from dead pigs for viral DNA detection using qPCR. The dashed black lines indicate the normal rectal temperature (40 °C) of pigs. LN1, inguinal lymph node; LN2, submaxillary lymph node; LN3, mediastinal lymph node. Source data are provided as a Source Data file.

In the 10^6^ HAD_50_ group, all six inoculated pigs started to have a fever on day 4 p.i. and died between day 5 p.i. and day 8 p.i. (Fig. 3). The two contact pigs started to have a fever on day 9 post-contact (p.ct.) and both died on day 12 p.ct. (Fig. 3). Viral DNA was detected in the oral swabs, rectal swabs, and blood of all inoculated pigs and contact pigs (Fig. 3). High levels of viral DNA were also detected in the organs, including the brain, heart, liver, spleen, lung, kidney, and tonsil, and three different lymph nodes (inguinal lymph node, submaxillary lymph node, and mediastinal lymph node) of the dead pigs (Fig. 3). In the 10^3^ HAD_50_ group, the inoculated pigs developed a fever on day 4 p.i., and all of the pigs died between day 6 p.i. and day 15 p.i. (Fig. 3). The two contact pigs started to develop a fever on day 9 p.ct. and day 10 p.ct., respectively; one died on day 12 p.ct. and the other died on day 14 p.ct. (Fig. 3). Viral DNA was also detected in the oral and rectal swabs, blood, and all the organs of all inoculated pigs and contact pigs, although the levels were slightly lower than those of the 10^6^ HAD_50_ group (Fig. 3). These results indicate that the recombinant virus JS/LG/21 is highly lethal and transmissible in pigs.

### The *MGF_505/360* genes and *EP402R* gene derived from Georgia07-like genotype II viruses contribute to the high virulence of the recombinant viruses in pigs

Many genes have been demonstrated to be important for the virulence of ASFV (Supplementary Table 4). Previous studies suggest that *MGF_505/360* and *EP402R* are important virulence factors for genotype I and II ASFVs^10,18,19^. The NH/P68-like genotype I viruses show low virulence in pigs^12,20^, whereas Georgia07-like genotype II viruses are highly lethal to pigs^15,19^. The genomes of the three recombinant isolates derive 43.5% of its fragments from NH/P68-like genotype I virus and 56.5% from Georgia07-like genotype II virus. However, the recombinant isolate showed the same lethality as the Georgia07-like genotype II virus in pigs. The *MGF_505/360* and *EP402R* genes are naturally defective in NH/P68-like viruses^21,22^. In this study, all three recombinant isolates contain the *MGF_505/360* genes and *EP402R* gene derived from the highly lethal Georgia07-like genotype II virus.

**Fig. 4:**
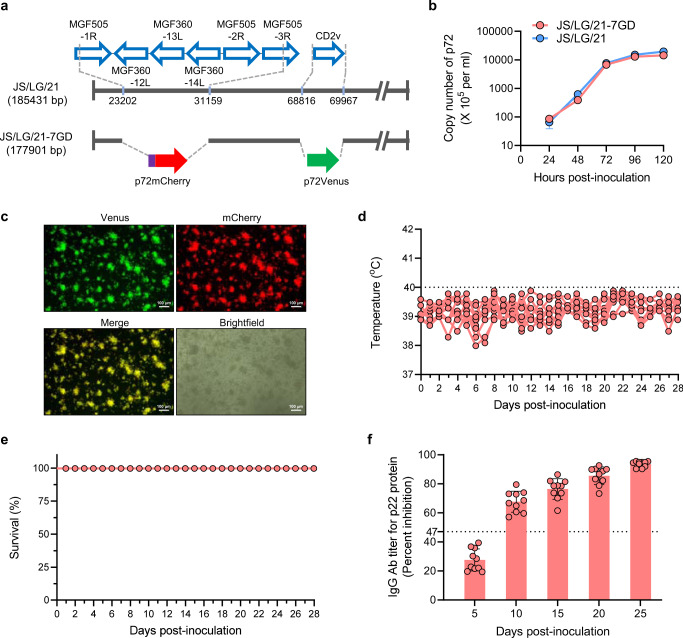
Virulence and transmissibility of JS/LG/21-7GD in Pigs. **a** Schematic representation of the gene-deleted JS/LG/21-7GD. The deleted gene segments were replaced with the p72mCherry and p72Venus reporter gene cassettes as indicated, respectively. Nucleotide positions indicating the boundaries of the deletion relative to the ASFV JS/LG/21 genome are indicated. **b** Growth curve of JS/LG/21 and JS/LG/21-7GD in PAMs. PAMs were infected with JS/LG/21 and JS/LG/21-7GD at an MOI of 0.1, respectively. The supernatants were collected from three wells for viral DNA detection using qPCR at the indicated timepoints. Data were presented as mean values ± SD. **c** Fluorescence of PAMs infected with JS/LG/21-7GD at 72 h post-inoculation. This experiment was performed three times and the data from one independent experiment were shown. Ten SPF pigs were inoculated with 10^6^ TCID_50_ of JS/LG/21-7GD. Rectal temperature (**d**) and survival (**e**) were monitored daily for 28 days after infection. **f** Sera were collected from ten inoculated pigs at the indicated timepoints for ASFV-specific antibody detection using a blocking ELISA kit (in-house). Data were presented as mean values ± SD. The dashed black lines indicate the normal rectal temperature (40 °C) of pigs. Source data are provided as a Source Data file.

To determine how these *MGF_505/360* and *EP402R* genes impact the virulence of the recombinant isolates, we generated the recombinant JS/LG/21 with deletion of *EP402R*, *MGF_505-1R*, *MGF_505-2R*, *MGF_505-3R*, *MGF_360-12L*, *MGF_360-13L*, and *MGF_360-14L* genes, and replaced these genes with the *Venus* and *mCherry* genes by using homologous recombination. The resultant virus was designed JS/LG/21-7GD (Fig. 4a). JS/LG/21-7GD had similar growth dynamics in PAMs to its parental virus JS/LG/21 (Fig. 4b), and the fluorescence of Venus and mCherry was observed in PAMs infected with JS/LG/21-7GD at 72 h post-infection (Fig. 4c). We tested the pathogenicity of JS/LG/21-7GD in SPF pigs by intramuscular inoculation at a dose of 10^6^ TCID_50_. All ten inoculated pigs had no fever and no ASFV-related clinical signs (Fig. 4d), survived the 28-day observation period (Fig. 4e), and developed substantial levels of ASFV-specific antibodies (Fig. 4f). These results suggest that the *MGF_505/360* genes and *EP402R* gene of genotype II virus are important virulence contributors to JS/LG/21.

### The recombinant virus JS/LG/21 can evade the immunity induced by a genotype II live attenuated ASFV vaccine

Previous studies have shown that protective immunity develops in pigs that survive a low-virulent ASFV infection, providing resistance to homologous virus challenge, but not to heterologous virus challenge^23,24^. A live attenuated vaccine strain HLJ/18-7GD was generated by deleting seven genes, including the six *MGF_505/360* genes and the *EP402R* gene that encodes CD2v, and studies in pigs have demonstrated the safety and efficacy of this vaccine against genotype II ASFV^10^. Could this candidate vaccine provide protection against the newly emerging recombinant virus? To answer this question, groups of five SPF pigs were intramuscularly vaccinated with 10^6^ TCID_50_ of HLJ/18-7GD, and then intramuscularly challenged with 10^3^ HAD_50_ of the recombinant virus JS/LG/21 or the genotype II virus HLJ/18 on day 28 post-vaccination. Groups of four unvaccinated pigs were challenged similarly as controls.

**Fig. 5:**
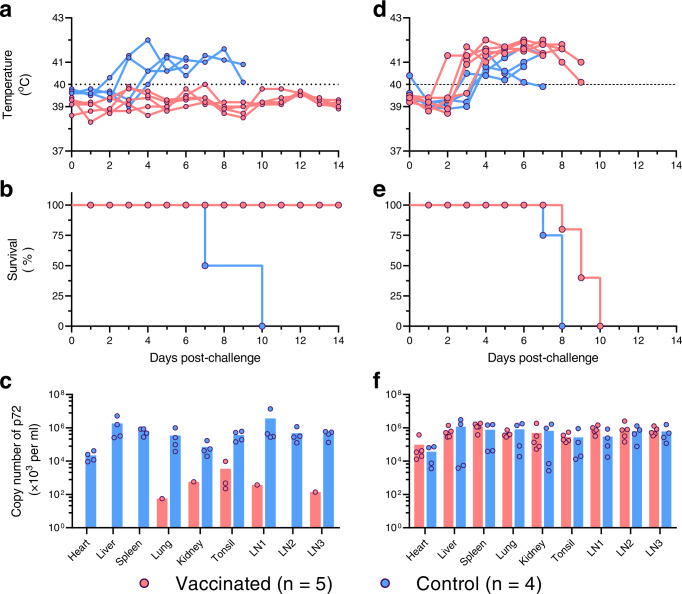
Protective efficacy of the HLJ/18-7GD vaccine against challenge with different ASFVs in pigs. Groups of five SPF pigs were vaccinated with 10^6^ TCID_50_ of HLJ/18-7GD, and then intramuscularly challenged with 10^3^ HAD_50_ of HLJ/18 (**a**–**c**) or the recombinant virus JS/LG/21 (**d**–**f**) on day 28 post-vaccination. Groups of four unvaccinated SPF pigs were challenged as controls. The rectal temperature (**a**, **d**) and survival (**b**, **e**) were monitored daily, and tissue samples (**c**, **f**) were collected from dead pigs or euthanized pigs at the end of the observation period for viral DNA detection using qPCR. The dashed black lines indicate the normal rectal temperature (40 °C) of pigs. LN1, inguinal lymph node; LN2, submaxillary lymph node; LN3, mediastinal lymph node. Source data are provided as a Source Data file.

Similar to our previous report^10^, the control pigs developed high fever and all died within 10 days of challenge with HLJ/18, and viral DNA was detected in the major organs and lymph nodes (Fig. 5). In contrast, the vaccinated pigs were well protected against the homologous virus HLJ/18 challenge: the pigs did not develop clinical symptoms and all of them survived the 28-day observation period (Fig. 5), although viral DNA was detected in the lung, kidney, and two lymph nodes of one pig and in the tonsil of three pigs that were euthanized at the end of the observation period (Fig. 5).The control pigs challenged with the recombinant virus JS/LG/21 developed fever and died within eight days of challenge, with virus detected in the major organs and lymph nodes (Fig. 5); however, we were surprised to find that all of the vaccinated pigs also developed fever and died within 10 days of challenge (Fig. 5), and the levels of viral replication in their organs were comparable to those in the control pigs (Fig. 5).These results indicate that the live attenuated vaccine HLJ/18-7GD cannot provide protection against the newly emerging recombinant ASFV.

## Discussion

In this study, we isolated three high lethal recombinant ASFVs with mosaic genomes composed of 56.5% Georgia07-like genotype II virus and 43.5% NH/P68-like genotype I virus. The recombinant virus is highly lethal and transmissible in pigs, and completely evades the immunity induced by vaccination with a Georgia07-like genotype II virus-based live attenuated vaccine.

The NH/P68-like genotype I virus SD/DY-I/21 detected in China is not lethal but causes clear necrotic skin lesions and joint swelling in pigs^12^. The low virulence phenotype of SD/DY-I/21-like genotype I virus is mainly due to the lack of the *MGF_505/360* genes in its genome and defective expression of the *EP402R* gene^12,13,25^. Twenty genes, including the *MGF_505/360* genes, *EP402R* gene, and *I177L* gene, have been demonstrated to be important for the virulence of genotype II ASFV^10,19,26–34^, and all these genes are in fragments F2, F6, or F20 of the recombinant viruses (Supplementary Table 4). Animal studies suggest that the *MGF_505/360* genes and *EP402R* gene play a key role in the highly lethal phenotype of the recombinant virus. How other virulent factors might contribute to the high pathogenicity of recombinant isolates remains to be determined in the future. Although the three recombinant viruses were isolated from pigs from three different provinces in China, the similar genomic structures suggest that the recombinant virus formed in one place and then spread, rather than the three recombinant viruses arising separately.

The genotype II ASFVs are currently widely circulating in pigs in many countries in Europe, Africa, and Asia^7,35^. Several gene-deleted live attenuated viruses based on the genotype II ASFVs have been generated by different groups and evaluated as potential vaccines^10,19,28–30,32,36,37^. The HLJ/18-7GD vaccine provides solid protection against the homologous genotype II virus, but does not provide any protection against the newly detected recombinant virus, indicating that the key elements in the genotype II virus targeted by the HLJ/18-7GD vaccine that induces protective immunity have been replaced by those of genotype I virus in the recombinant virus. The lack of protection might also be the result of the superposition effect of antigenic deviation of genotype I and the immune evasion function of genes derived from lethal genotype II virus^28,33,34^. Previous studies have shown that attenuated genotype I ASFV vaccine candidates could induce cross-protection against virulent genotype II virus challenge^18,38,39^. Hence, it is worth evaluating whether other attenuated vaccines induce protection against these emerging recombinants of genotype I and II viruses. Our preliminary data showed that JS/LG/21-7GD was highly attenuated in pigs, and thus the potential of JS/LG/21-7GD to be a safe and effective vaccine against virulent recombinant viruses and their parent-like genotype I and II viruses should be evaluated further. To date, progress on unraveling the fundamental mechanism of immune protection against ASFV has been limited, which has, in turn, hindered progress on vaccine research and development. The recombinant JS/LG/21 completely evades the protective immunity induced by the genotype II live attenuated vaccine candidate HLJ/18-7GD, which provides us with very important clues and useful information to explore the protection mechanism for ASFVs.

In summary, here we detected naturally occurring recombinants of genotype I and genotype II ASFVs in pigs in China. Our animal studies indicate that these viruses are highly lethal and transmissible in pigs and that the genotype II virus-based live attenuated vaccine cannot provide protection against these recombinant viruses.

## Methods

### Facility and ethics statements

All experiments with live ASFVs were performed in the BSL-3/ABSL-3 biosafety facilities at the Harbin Veterinary Research Institute (HVRI) of the Chinese Academy of Agricultural Sciences (CAAS) as approved by the Ministry of Agriculture and Rural Affairs. This study was performed in accordance with the Guide for the Care and Use of Laboratory Animals of the Ministry of Science and Technology of the People’s Republic of China. The protocols were approved by the Committee on the Ethics of Animal Experiments of the HVRI of CAAS (Approval numbers: 220401-03-GJ, 220421-03-GJ, and 221027-02-GJ).

### Cells and viruses

Primary porcine alveolar macrophages (PAMs) and peripheral blood mononuclear cells (PBMCs) were prepared from the bronchoalveolar lavage and EDTA-treated blood of 30-day-old specific-pathogen-free (SPF) pigs as previously described^15^. Blood or homogenized tissue samples, including lung, spleen, and lymph nodes, were used to inoculate PAMs. On day 5 post-inoculation (p.i.), the cell supernatants were examined in a qPCR targeting the *B646L* gene of ASFVs. ASFV-positive cell supernatants were collected and virus purification was performed in PAMs by three rounds of limiting dilution. The ASFV isolate was titrated in PAMs by using an immunofluorescence assay or hemadsorption assay, and determined to be free of contamination with bacteria, porcine circovirus type 2, pseudorabies virus, porcine respiratory and reproductive syndrome virus, or classical swine fever virus. The recombinant virus with deletion of *EP402R*, *MGF_505-1R*, *MGF_505-2R*, *MGF_505-3R*, *MGF_360-12L*, *MGF_360-13L*, and *MGF_360-14L* genes was generated by using homologous recombination as previously described^10^. All virus stock was aliquoted and stored at −80 °C.

### Hemadsorption (HAD) assay

The HAD activity of each ASFV was assessed as previously described in ref. ^15^. Briefly, tenfold serially diluted virus solutions were inoculated into PBMCs with 0.1% porcine red blood cells in 96-well plates, respectively. HAD was observed daily for 7 days after inoculation under a microscope. The 50% HAD dose (HAD_50_) for each virus was measured using the Reed and Muench method^40^.

### qPCR for virus detection

ASFV genomic DNA in swabs, tissue homogenates, serum, EDTA-treated whole peripheral blood samples, and cell supernatants was extracted using QIAamp® DNA Mini Kits (Qiagen, Germany) and detected using WOAH-recommended qPCR as described previously in refs. ^11, 41^.

### Genome sequencing and genetic analysis

ASFV genome segments were amplified by PCR using specific primers and a high-fidelity reaction system and sequenced using the Sanger DNA sequencing method as described previously in refs. ^11, 12^. To construct the phylogenetic tree, all ASFV genomes were aligned using E-INS-i of the program MAFFT v7^42^ and the ambiguously aligned regions were excluded using Gblocks-0.91^43^. Phylogenetic analysis was inferred using maximum likelihood (ML), and ModelFinder was used to select the best-fit model according to the Bayesian information criterion (BIC)^44^. ML analysis was inferred using IQ-Tree^45^ and the best model was GTR + F + R3. Bootstrap branch support values (MLBS) were obtained with 1000 rapid bootstrap inferences and subsequently sought in a thorough ML search of the dataset. The diagram of the ASFV genome was created using the BLAST Ring Image Generator (BRIG) with the whole genome sequence of JS/LG/21. The BLAST option of BRIG was ncbi-blast-2.13.0+. Different recombination regions and the open reading frames (ORFs) were analyzed in comparison with those of genotype II virus HLJ/18 and genotype I virus SD/DY-I/21, respectively. Biological software, including Snapgene® 4.1.8 and MEGA X (https://www.megasoftware.net), were used for the alignment of multiple sequences. Primer sequences for PCR amplification and sequencing used in this study are shown in Supplementary Dataset 2.

### Pig study

To evaluate the virulence of ASFV in pigs, groups of 7-week-old SPF Landrace pigs, regardless of sex, from the Laboratory Animal Center of HVRI were intramuscularly (i.m.) inoculated with 10^6^ HAD_50_ or 10^3^ HAD_50_ of JS/LG/21. Two additional naïve SPF pigs were then cohoused with the infected pigs in each group from the first day of infection to evaluate the transmission of JS/LG/21. All pigs were monitored daily for survival and clinical signs. Oral swabs, rectal swabs, and EDTA-treated blood were collected for virus DNA quantification using qPCR at the indicated times p.i. or post-contact (p.ct.). Organs and lymph nodes were collected from dead or euthanized pigs and examined for viral DNA using qPCR as described previously^11,41^.

To examine the virulence of JS/LG/21-7GD in pigs, ten 7-week-old SPF Landrace pigs, regardless of sex, were i.m. inoculated with 10^6^ TCID_50_ of JS/LG/21-7GD. All pigs were monitored daily for rectal temperature, survival, and clinical signs for 28 days p.i. Pig sera were collected at the indicated timepoints for ASFV-specific antibody detection using a blocking ELISA kit (in-house)^46^.

To evaluate the protective efficacy of genotype II live attenuated vaccine HLJ/18-7GD against the new emerging ASFV isolates, groups of 7-week-old SPF Landrace pigs regardless of sex were *i.m*. inoculated with 10^6^ TCID_50_ of HLJ/18-7GD, and were then *i.m*. challenged with 10^3^ HAD_50_ of JS/LG/21 or HLJ/18 on day 28 post-vaccination (p.v.). Groups of four unvaccinated pigs were parallelly challenged as controls. All pigs were daily monitored for rectal temperature, survival, and clinical signs for 28 days post-challenge (p.ch.). Organ tissues, including heart, liver, spleen, lung, kidney, tonsil, and lymph nodes, were collected from the dead pigs or surviving pigs euthanized at the end of the observation period to detect virus loading using qPCR.

### Reporting summary

Further information on research design is available in the Nature Portfolio Reporting Summary linked to this article.

## Supplementary information

Supplementary Information

Description of Additional Supplementary Files

Supplementary Data 1

Supplementary Data 2

Reporting Summary

## Data Availability

All data were available in the manuscript or the supplementary data. Source data are provided with this paper. Sequences of the viruses used in this study have been deposited in GenBank (accession numbers: OQ504954 for HeN/123014/22, OQ504955 for IM/DQDM/22, and OQ504956 for JS/LG/21). Source data are provided with this paper.

## Source data

Source Data

## Code availability

This study did not use any custom code or mathematical algorithm.
