# Supplementary material for: Highly lethal genotype I and II recombinant African swine fever viruses detected in pigs
Source: Nat Commun. 2023 May 29;14:3096. doi: 10.1038/s41467-023-38868-w (PMC10226439; doi:10.1038/s41467-023-38868-w)
Supplement: Supplementary file 1 — Supplementary Information [file 41467_2023_38868_MOESM1_ESM.pdf]

Supplementary Information

Highly lethal genotype I and II recombinant African swine fever viruses detected in pigs

Dongming Zhao[1]*, Encheng Sun[1]*, Lianyu Huang[1]*, Leilei Ding[1]*, Yuanmao Zhu[1]*, Jiwen Zhang[1], Dongdong Shen[1], Xianfeng Zhang[1], Zhenjiang Zhang[1], Tao Ren[1], Wan Wang[1], Fang Li[1], Xijun He[1], Zhigao Bu[1,2]†

[1]State Key Laboratory for Animal Disease Control and Prevention, National High Containment Facilities for Animal Diseases Control and Prevention, Harbin Veterinary Research Institute, Chinese Academy of Agricultural Sciences, Harbin, People's Republic of China.

[2]Jiangsu Co-innovation Center for Prevention and Control of Important Animal Infectious Diseases and Zoonoses, Yangzhou University, Yangzhou, People's Republic of China.

*These authors equally contributed to this study.

†Corresponding author. Email: buzhigao@caas.cn

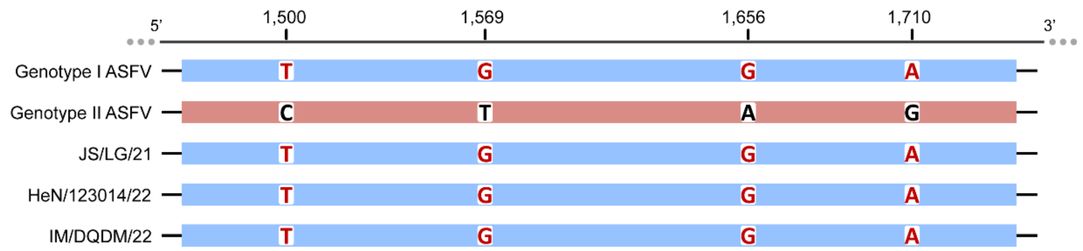

Supplementary Figure 1. Comparison of partial sequences of the *B646L* gene at its C-terminus. The 415-nt C-terminal regions of the *B646L* ORF from different ASFVs were aligned using MEGA X (https://www.megasoftware.net). Sites where the nucleotides differ are shown based on the full-length *B646L* ORF sequence from genotype I virus SD/DY-I/21.

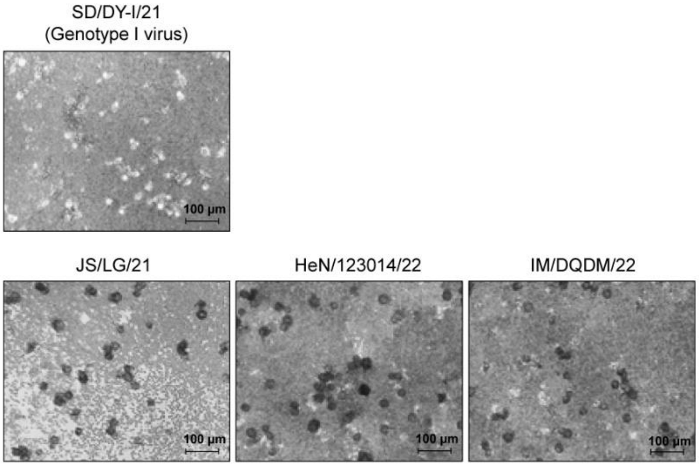

Supplementary Figure 2. Hemadsorption (HAD) phenotypes of different ASFVs in PBMCs. The indicated viruses were 10-fold serially diluted, and then inoculated into PBMCs with 0.1% porcine red blood cells in 96-well plates. The HAD phenotype was daily observed under a microscope and recorded for 7 days after inoculation. This experiment was performed three times and the data from one independent experiment were shown.

Supplementary Table 1. Homology of each fragment of the recombinant African swine fever viruses (ASFVs) with the corresponding fragment of genotype I and genotype II ASFVs.

Strain	Fragment					Identity (%) with that of	
	ID	Position		Length (bp)	Origin	SD/DY-I/21	HLJ/18
		Start	End				
HeN/123014/22	F1	1	19,267	19,267	Genotype I	99.88	71.28
	F2	19,268	33,242	13,975	Genotype II	27.50	99.99
	F3	33,243	59,372	26,130	Genotype I	99.99	93.71
	F4	59,373	63,488	4,116	Genotype II	96.55	100.00
	F5	63,489	66,702	3,214	Genotype I	100.00	95.41
	F6	66,307	77,556	11,250	Genotype II	89.57	100.00
	F7	77,557	81,248	3,692	Genotype I	99.89	97.49
	F8	81,249	86,877	5,629	Genotype II	96.96	99.96
	F9	86,878	102,483	15,606	Genotype I	99.36	95.21
	F10	102,484	115,806	13,323	Genotype II	93.73	100.00
	F11	115,807	118,769	2,963	Genotype I	99.93	90.09
	F12	118,770	128,914	10,145	Genotype II	96.01	99.96
	F13	128,915	131,695	2,781	Genotype I	99.93	97.94
	F14	131,696	136,759	5,064	Genotype II	98.28	99.96
	F15	136,760	137,307	548	Genotype I	100.00	98.91
	F16	137,308	140,815	3,508	Genotype II	97.97	100.00
	F17	140,816	142,214	1,399	Genotype I	99.71	99.15
	F18	142,215	149,844	7,630	Genotype II	97.03	100.00
	F19	149,845	154,884	5,040	Genotype I	99.96	98.17
	F20	154,885	185,395	30,511	Genotype II	85.60	99.95
IM/DQDM/22	F1	1	19,275	19,275	Genotype I	99.90	71.30
	F2	19,276	33,250	13,975	Genotype II	27.50	99.99
	F3	33,251	59,380	26,130	Genotype I	99.99	93.72
	F4	59,381	63,496	4,116	Genotype II	96.55	100.00
	F5	63,497	66,710	3,214	Genotype I	100.00	95.41
	F6	66,711	77,564	10,854	Genotype II	89.57	100.00
	F7	77,565	81,256	3,692	Genotype I	99.89	97.49
	F8	81,257	86,885	5,629	Genotype II	96.96	99.96
	F9	86,886	102,491	15,606	Genotype I	99.36	95.21
	F10	102,492	115,814	13,323	Genotype II	93.73	100.00
	F11	115,815	118,777	2,963	Genotype I	100.00	90.15
	F12	118,778	128,922	10,145	Genotype II	96.03	99.98
	F13	128,923	131,703	2,781	Genotype I	100.00	98.01
	F14	131,704	136,767	5,064	Genotype II	98.32	100.00
	F15	136,768	137,315	548	Genotype I	100.00	98.91
	F16	137,316	140,823	3,508	Genotype II	97.97	100.00
	F17	140,824	142,222	1,399	Genotype I	99.71	99.15
	F18	142,223	149,852	7,630	Genotype II	97.03	100.00
	F19	149,853	154,892	5,040	Genotype I	100.00	98.21
	F20	154,893	185,342	30,450	Genotype II	85.61	100.00

Supplementary Table 2. Changes in the open reading frame (ORF) of the genotype I virus-derived fragments of the three recombinant African swine fever viruses compared with that of SD/DY-I/21.

**ORF**	**Length [number of amino acids (AAs)] in SD/DY-I/21**	**JS/LG/21**		**HeN/123014/22**		**IM/DQDM/22**	
		**Length**	**Change**	**Length**	**Change**	**Length**	**Change**
MGF_110-13L	163	13	150 AAs deleted at the C-terminus due to the deletion of a C at position 11,609	13	150 AAs deleted at the C-terminus due to the deletion of a C at position 11,609.	163	/
B602L	638	658	32 AAs inserted at position 172 due to the insertion of 96 nucleotides at position 87,589 and 12 AAs deleted from positions 237 to 248 due to the deletion of 36 nucleotides from positions 87,361 to 87,396.	670	32 AAs inserted at position 172 due to the insertion of 96 nucleotides at position 87,589.	670	32 AAs inserted at position 172 due to the insertion of 96 nucleotides at position 87,589.

Supplementary Table 3. Changes in the open reading frame (ORF) of the genotype II virus-derived fragments of the three recombinant African swine fever viruses compared with that of HLJ/18.

**ORF**	**Length [number of amino acids (AAs)] in HLJ/18**	**JS/LG/21**		**HeN/123014/22**		**IM/DQDM/22**	
		**Length**	**Change**	**Length**	**Change**	**Length**	**Change**
MGF_360-14L	357	287	70 AAs deleted at the C-terminus due to the insertion of a C at position 32,082.	287	70 AAs deleted at the C-terminus due to the insertion of a C at position 32,082.	287	70 AAs deleted at the C-terminus due to the insertion of a C at position 32,082.
I9R	96	103	Seven AAs inserted at the C-terminus due to the insertion of an A at position 182,024.	103	Seven AAs inserted at the C-terminus due to the insertion of an A at position 182,024.	103	Seven AAs inserted at the C-terminus due to the insertion of an A at position 182,024.

Supplementary Table 4. Virulence-related genes in the genotype II virus-derived genome fragments of the recombinant African swine fever viruses.

Fragment	Virulence-related genes	Reference
**F2**	*MGF_360-9L*	[1]
	MGF_505-1R, MGF_360-12L, MGF_360-13L, MGF_360-14L, MGF_505-2R, MGF_505-3R	[2-6]
**F6**	*EP153R*	[7,8]
	EP402R	[6-8]
**F20**	*QP509L, QP383R*	[9]
	E184L	[10]
	I267L	[11]
	I226R	[12]
	I177L	[13]
	I7L, I8L, I9R, I10L, L11L	[14]

Supplementary References:

1 Zhang, K. *et al.* MGF360-9L Is a Major Virulence Factor Associated with the African Swine Fever Virus by Antagonizing the JAK/STAT Signaling Pathway. *mBio*, e0233021, doi:10.1128/mbio.02330-21 (2022).

2 Rathakrishnan, A. *et al.* Differential Effect of Deleting Members of African Swine Fever Virus Multigene Families 360 and 505 from the Genotype II Georgia 2007/1 Isolate on Virus Replication, Virulence, and Induction of Protection. *Journal of virology* **96**, e0189921, doi:10.1128/jvi.01899-21 (2022).

3 O'Donnell, V. *et al.* African swine fever virus Georgia isolate harboring deletions of 9GL and MGF360/505 genes is highly attenuated in swine but does not confer protection against parental virus challenge. *Virus research* **221**, 8-14, doi:10.1016/j.virusres.2016.05.014 (2016).

4 O'Donnell, V. *et al.* African Swine Fever Virus Georgia Isolate Harboring Deletions of MGF360 and MGF505 Genes Is Attenuated in Swine and Confers Protection against Challenge with Virulent Parental Virus. *Journal of virology* **89**, 6048-6056, doi:10.1128/JVI.00554-15 (2015).

5 Reis, A. L. *et al.* Deletion of African swine fever virus interferon inhibitors from the genome of a virulent isolate reduces virulence in domestic pigs and induces a protective response. *Vaccine* **34**, 4698-4705, doi:10.1016/j.vaccine.2016.08.011 (2016).

6 Chen, W. *et al.* A seven-gene-deleted African swine fever virus is safe and effective as a live attenuated vaccine in pigs. *Sci China Life Sci* **63**, 623-634, doi:10.1007/s11427-020-1657-9 (2020).

7 Petrovan, V. *et al.* Role of African Swine Fever Virus Proteins EP153R and EP402R in Reducing Viral Persistence in Blood and Virulence in Pigs Infected with BeninDeltaDP148R. *Journal of virology* **96**, e0134021, doi:10.1128/JVI.01340-21 (2022).

8 Gladue, D. P. *et al.* Deletion of CD2-Like (CD2v) and C-Type Lectin-Like (EP153R) Genes from African Swine Fever Virus Georgia-9GL Abrogates Its Effectiveness as an Experimental Vaccine. *Viruses* **12**, doi:10.3390/v12101185 (2020).

9 Li, D. *et al.* A QP509L/QP383R-Deleted African Swine Fever Virus Is Highly Attenuated in Swine but Does Not Confer Protection against Parental Virus Challenge. *Journal of virology* **96**, e0150021, doi:10.1128/JVI.01500-21 (2022).

10 Ramirez-Medina, E. *et al.* Deletion of E184L, a Putative DIVA Target from the Pandemic Strain of African Swine Fever Virus, Produces a Reduction in Virulence and Protection against Virulent Challenge. *Journal of virology* **96**, e0141921, doi:10.1128/JVI.01419-21 (2022).

11 Ran, Y. *et al.* African swine fever virus I267L acts as an important virulence factor by inhibiting RNA polymerase III-RIG-I-mediated innate immunity. *PLoS pathogens* **18**, e1010270, doi:10.1371/journal.ppat.1010270 (2022).

12 Zhang, Y. *et al.* African Swine Fever Virus Bearing an I226R Gene Deletion Elicits Robust Immunity in Pigs to African Swine Fever. *Journal of virology* **95**, e0119921, doi:10.1128/JVI.01199-21 (2021).

13 Borca, M. V. *et al.* Development of a Highly Effective African Swine Fever Virus Vaccine by Deletion of the I177L Gene Results in Sterile Immunity against the Current Epidemic Eurasia Strain. *Journal of virology* **94**, doi:10.1128/JVI.02017-19 (2020).

14 Zhang, J. *et al.* Deletion of the L7L-L11L Genes Attenuates ASFV and Induces Protection against Homologous Challenge. *Viruses* **13**, doi:10.3390/v13020255 (2021).
