# Supplementary material for: Highly lethal genotype I and II recombinant African swine fever viruses detected in pigs
Source: Nat Commun. 2023 May 29;14:3096. doi: 10.1038/s41467-023-38868-w (PMC10226439; doi:10.1038/s41467-023-38868-w)
Supplement: Supplementary file 2 — Description of Additional Supplementary Files [file 41467_2023_38868_MOESM2_ESM.pdf]

Description of Additional Supplementary Files

Supplementary Data 1. Information of the recombinant regions derived from genotype I and II ASFVs in the genomes of the three recombinants.

Supplementary Data 2. The information of primers for PCR amplification and sequencing of viral genomes.
