# Supplementary material for: Highly lethal genotype I and II recombinant African swine fever viruses detected in pigs
Source: Nat Commun. 2023 May 29;14:3096. doi: 10.1038/s41467-023-38868-w (PMC10226439; doi:10.1038/s41467-023-38868-w)
Supplement: Supplementary file 5 — Reporting Summary [file 41467_2023_38868_MOESM5_ESM.pdf]

nature portfolio

Corresponding author(s): Zhigao Bu

Last updated by author(s): Apr 6, 2023

Reporting Summary

Nature Portfolio wishes to improve the reproducibility of the work that we publish. This form provides structure for consistency and transparency in reporting. For further information on Nature Portfolio policies, see our Editorial Policies and the Editorial Policy Checklist.

Statistics

For all statistical analyses, confirm that the following items are present in the figure legend, table legend, main text, or Methods section.

n/a	Confirmed	
☐	☒	The exact sample size (*n*) for each experimental group/condition, given as a discrete number and unit of measurement
☒	☐	A statement on whether measurements were taken from distinct samples or whether the same sample was measured repeatedly
☒	☐	The statistical test(s) used AND whether they are one- or two-sided<br>*Only common tests should be described solely by name; describe more complex techniques in the Methods section.*
☐	☒	A description of all covariates tested
☒	☐	A description of any assumptions or corrections, such as tests of normality and adjustment for multiple comparisons
☒	☐	A full description of the statistical parameters including central tendency (e.g. means) or other basic estimates (e.g. regression coefficient) AND variation (e.g. standard deviation) or associated estimates of uncertainty (e.g. confidence intervals)
☒	☐	For null hypothesis testing, the test statistic (e.g. *F*, *t*, *r*) with confidence intervals, effect sizes, degrees of freedom and *P* value noted<br>*Give P values as exact values whenever suitable.*
☒	☐	For Bayesian analysis, information on the choice of priors and Markov chain Monte Carlo settings
☒	☐	For hierarchical and complex designs, identification of the appropriate level for tests and full reporting of outcomes
☒	☐	Estimates of effect sizes (e.g. Cohen's *d*, Pearson's *r*), indicating how they were calculated

Our web collection on statistics for biologists contains articles on many of the points above.

Software and code

Policy information about availability of computer code

Data collection	No software was used.
Data analysis	DNASTAR Lasergene 7; MAFFT v7; Gblocks-0.91; IQ-Tree 2.0; ModelFinder (included in IQ-Tree); SnapGene 4.1.8; MEGA X; BLAST Ring Image Generator （BRIG) (https://brig.sourceforge.net/), BRIG-0.95-dist， BLAST option: ncbi-blast-2.13.0+

For manuscripts utilizing custom algorithms or software that are central to the research but not yet described in published literature, software must be made available to editors and reviewers. We strongly encourage code deposition in a community repository (e.g. GitHub). See the Nature Portfolio guidelines for submitting code & software for further information.

Data

Policy information about availability of data

All manuscripts must include a data availability statement. This statement should provide the following information, where applicable:

- Accession codes, unique identifiers, or web links for publicly available datasets
- A description of any restrictions on data availability
- For clinical datasets or third party data, please ensure that the statement adheres to our policy

All data are available in the manuscript or the supplementary data. Source data are provided with this paper. Sequences of the viruses used in this study have been deposited in GenBank (accession numbers: OQ504954 for HeN/123014/22, OQ504955 for IM/DQDM/22, and OQ504956 for JS/LG/21). Genotype II virus HLJ/18 (GenBank: MK333180) and genotype I virus SD/DY-I/21 (GenBank: MZ945537) are available in GenBank.

Human research participants

Policy information about studies involving human research participants and Sex and Gender in Research.

Reporting on sex and gender	No human research
Population characteristics	See above.
Recruitment	No human research
Ethics oversight	No human research

Note that full information on the approval of the study protocol must also be provided in the manuscript.

Field-specific reporting

Please select the one below that is the best fit for your research. If you are not sure, read the appropriate sections before making your selection.

☒ Life sciences ☐ Behavioural & social sciences ☐ Ecological, evolutionary & environmental sciences

For a reference copy of the document with all sections, see nature.com/documents/nr-reporting-summary-flat.pdf

Life sciences study design

All studies must disclose on these points even when the disclosure is negative.

Sample size	All sample sizes are more than 3, because more than 3 numbers can be used for statistic analysis.
Data exclusions	No data were excluded from the analyses.
Replication	Generally the experiments were performed three times and the data from one independent experiment were shown. For animal studies, just performed once without replication.
Randomization	The same aged pigs were randomly divided into different groups for experiments.
Blinding	The investigators were blinded to group allocation during data collection and analysis.

Reporting for specific materials, systems and methods

We require information from authors about some types of materials, experimental systems and methods used in many studies. Here, indicate whether each material, system or method listed is relevant to your study. If you are not sure if a list item applies to your research, read the appropriate section before selecting a response.

Materials & experimental systems

n/a	Involved in the study	
☒	☐	Antibodies
☒	☐	Eukaryotic cell lines
☒	☐	Palaeontology and archaeology
☐	☒	Animals and other organisms
☒	☐	Clinical data
☒	☐	Dual use research of concern

Methods

n/a	Involved in the study	
☒	☐	ChIP-seq
☒	☐	Flow cytometry
☒	☐	MRI-based neuroimaging

Animals and other research organisms

Policy information about studies involving animals; ARRIVE guidelines recommended for reporting animal research, and Sex and Gender in Research

Laboratory animals	7-week-old specific-pathogen-free Landrace pigs
Wild animals	The study did not involve wild animals.

Reporting on sex	Sex was not considered in study design.
Field-collected samples	The blood or lymph nodes from the pigs in pig farms or slaughter houses were collected and packed following biosafety regulation, and were delivered to the lab at 4 degree.
Ethics oversight	All experiments with live ASFVs were performed in the BSL-3/ABSL-3 biosafety facilities at the Harbin Veterinary Research Institute (HVRI) of the Chinese Academy of Agricultural Sciences (CAAS) as approved by the Ministry of Agriculture and Rural Affairs. This study was performed in accordance with the Guide for the Care and Use of Laboratory Animals of the Ministry of Science and Technology of the People's Republic of China. The protocols were approved by the Committee on the Ethics of Animal Experiments of the HVRI of CAAS (Approval numbers: 220401-03-GJ, 220421-03-GJ, and 221027-02-GJ).

Note that full information on the approval of the study protocol must also be provided in the manuscript.
